# Uncovering the Transcriptional Correlates of Hub Connectivity in Neural Networks

**DOI:** 10.3389/fncir.2019.00047

**Published:** 2019-07-19

**Authors:** Aurina Arnatkevičiūtė, Ben D. Fulcher, Alex Fornito

**Affiliations:** ^1^Monash Biomedical Imaging, School of Psychological Sciences, Turner Institute for Brain and Mental Health, Monash University, Clayton, VIC, Australia; ^2^School of Physics, The University of Sydney, Sydney, NSW, Australia

**Keywords:** connectome, hub, rich-club, gene expression, network neuroscience, graph theory, genome

## Abstract

Connections in nervous systems are disproportionately concentrated on a small subset of neural elements that act as network hubs. Hubs have been found across different species and scales ranging from *C. elegans* to mouse, rat, cat, macaque, and human, suggesting a role for genetic influences. The recent availability of brain-wide gene expression atlases provides new opportunities for mapping the transcriptional correlates of large-scale network-level phenotypes. Here we review studies that use these atlases to investigate gene expression patterns associated with hub connectivity in neural networks and present evidence that some of these patterns are conserved across species and scales.

## 1. Introduction

The brain is a multiscale network, with neuronal elements exhibiting coordinated patterns of activity that unfold across several orders of magnitude in time and space (Buzsáki and Draguhn, 2004; Lichtman and Denk, 2011; Fornito et al., 2016). Graph theory provides a useful approach to represent network organization at each scale by focusing on the essential elements of the system: processing units and their interactions, represented, respectively, as nodes and edges in the graph (Bullmore and Sporns, 2009; Fornito et al., 2016). The advantage of using a graph theoretic approach to understand the organizational properties of the brain is that the same analysis tools can be applied regardless of the species or scale, ranging from electron micrograph data of neuron-and-synapse connectivity in the nematode worm *Caenorhabditis elegans* (White et al., 1986; Varshney et al., 2011), through tract-tracing data in the mouse (Oh et al., 2014; Gămănuţ et al., 2018) and macaque (Stephan et al., 2001; Markov et al., 2014), to brain-wide non-invasive structural and functional imaging in the human (Bassett and Bullmore, 2009; Bullmore and Sporns, 2009; Fornito et al., 2013).

A growing body of work has demonstrated that the connection topology of neural networks—that is, the specific arrangement of connections between system elements—shows a number of non-random properties that are conserved across different scales and in different species (Bullmore and Sporns, 2009; Sporns, 2011; Fornito et al., 2016; van den Heuvel et al., 2016a; Schröter et al., 2017). These include (i) a predominance of short-range, locally clustered connections supporting functional specialization coupled with sparse, long-range projections that may promote global integration and functional diversity, resulting in an economical small-world organization (Watts and Strogatz, 1998; Bassett and Bullmore, 2017; Betzel and Bassett, 2017); (ii) the presence of densely connected sub-networks, termed modules, organized hierarchically across several resolution levels so that modules contain nested sub-modules and so on (Meunier et al., 2009; Bassett et al., 2010); (iii) a fat-tailed distribution of connectivity across nodes, such that some nodes possess a relatively large number of connections and act as network hubs (van den Heuvel and Sporns, 2011; Towlson et al., 2013; van den Heuvel et al., 2016a); and (iv) a dense inter-connectivity of hub nodes, leading to the formation of a “rich-club” (Zamora-López et al., 2010; van den Heuvel and Sporns, 2011; Harriger et al., 2012; Towlson et al., 2013).

The strong conservation of such topological properties across scales and species implies that particular connectivity patterns are being evolutionary favored either through common descent or convergent evolutionary paths. This raises questions concerning the degree to which genes influence brain network topology. Twin studies have shown that topological properties of human brain networks mapped at the macroscale are heritable (Smit et al., 2008; Fornito et al., 2011; van den Heuvel et al., 2013a; Bohlken et al., 2014; Sinclair et al., 2015; Zhan et al., 2015; Colclough et al., 2017), but they do not indicate the specific genes involved. Studies linking structural variation in the genome to variability in network-level phenotypes, both at the level of candidate genes (Liu et al., 2010; Brown et al., 2011; Dennis et al., 2011; Markett et al., 2017) and in genome-wide scans (Jahanshad et al., 2013), have started to address this gap. However, they provide a partial picture, as it is often unclear how a given DNA variant impacts gene expression to give rise to phenotypic variability.

In neuroscience, it has been difficult to link direct measures of gene expression to variation in network phenotypes defined across large swathes of the brain, as gene expression has traditionally only been quantifiable though invasive interrogation of regionally localized tissue samples. The recent availability of large-scale, brain-wide atlases of gene expression (Lein et al., 2007; Hawrylycz et al., 2012), has overcome this hurdle and presented new opportunities to understand the molecular correlates of network-level phenotypes. Patterns of gene expression have been used to predict whether two neurons (or large-scale brain regions) will be structurally connected (Kaufman et al., 2006; Varadan et al., 2006; Baruch et al., 2008; French and Pavlidis, 2011; Wolf et al., 2011; Ji et al., 2014; Fakhry and Ji, 2015), and confirmed that regional variations in gene expression track specific aspects of structural (Goel et al., 2014; Forest et al., 2017; Parkes et al., 2017; Romero-Garcia et al., 2018) and functional (Cioli et al., 2014; Hawrylycz et al., 2015; Richiardi et al., 2015; Krienen et al., 2016; Anderson et al., 2018) brain networks. The integration of gene expression atlases with imaging data is also shedding light on the molecular correlates of macroscopic brain changes observed in a range of disorders, such as Huntington's disease (McColgan et al., 2018), Parkinson's disease (Rittman et al., 2016), and schizophrenia (Romme et al., 2017).

One important aspect of brain network organization is the distribution of connections across nodes, which is disproportionately concentrated on a small number of network hubs (van den Heuvel and Sporns, 2011; Towlson et al., 2013). Most simply, network hubs are defined as nodes with a relatively large number of connections, placing them in a topologically central position within the network (although other definitions are possible; see Power et al., 2011; Oldham et al., 2018). Intuitively, the global air transportation network offers insight into the role of hubs in mediating network traffic flow; certain airports, such as Dubai International, London Heathrow, and LAX are linked to the rest of the network by a much larger number of direct flights than other airports. They are thus positioned to mediate a large fraction of intercontinental travel. Similarly, connections are not distributed equally across neurons, neuronal populations or large brain areas, with specific network elements possessing the lion's share of connections (van den Heuvel and Sporns, 2011; Towlson et al., 2013; de Reus and van den Heuvel, 2014; van den Heuvel et al., 2016a). These brain hubs are thought to play a critical role in the functional integration of anatomically disparate systems (Harriger et al., 2012; van den Heuvel et al., 2012), and are disproportionately impacted by a diverse variety of brain diseases (Crossley et al., 2014; Fornito et al., 2015). Thus, understanding the molecular basis for hub connectivity may provide insights not only into integrated cerebral function, but also into the various disease processes that plague the brain.

In this article, we review how brain-wide gene expression atlases have been used to link two traditionally disparate scales of analysis in neuroscience: molecular function (microscale) and whole-brain network topology (macroscale), by identifying the transcriptional correlates of brain network hubs. We begin with a brief overview of the expression atlases that are currently available and then consider how hubs are defined in brain networks and what we know about their functional role. We then examine research indicating that brain network hubs possess a distinct and conserved transcriptional signature.

## 2. Characterizing Gene Expression Across the Entire Brain

Gene expression is a process through which genetic information encoded in sequences of DNA is read and used to synthesize a particular gene product. The two key steps in this complex process are transcription, where an unwound segment of DNA is read to produce messenger (mRNA), and translation, which occurs when the resulting mRNA is used to synthesize the gene product, such as a protein. Gene expression is commonly inferred from mRNA levels, thus serving as an index of transcriptional activity—an indirect proxy for the protein abundance. Transcriptional activity can be measured using several different techniques that either assay bulk tissue samples [microarray (Schulze and Downward, 2001), RNA-seq (Mortazavi et al., 2008; Wang et al., 2009)], histological sections at a cellular resolution [*in situ* hybridization (ISH) (Schulze and Downward, 2001)], or single cells [single-cell RNA sequencing (scRNA-seq) (Tang et al., 2009)]. Different classes of brain cells show distinctive gene expression patterns (Darmanis et al., 2015; Poulin et al., 2016; Tasic et al., 2016; Mancarci et al., 2017), and scRNA-seq is thus regarded as the most promising technology for accurately resolving cell-type specificity (Yu and Lin, 2016). However, scRNA-seq is difficult to scale to brain-wide analyses, and current brain-wide atlases of gene expression have relied on microarray or ISH. ISH has high spatial resolution, allowing gene expression to be measured in a tissue section with relatively high sensitivity and specificity, but requires a very large number of samples to quantify expression levels across thousands of genes (Unger et al., 2010). ISH has therefore only been used to construct atlases for species with high tissue availability, such as the mouse (Lein et al., 2007). Microarray, on the other hand, allows the quantification of expression levels of thousands of genes at once by measuring the hybridization of cRNA (Cy3-labeled RNA) in a tissue sample to particular spot (probe) on the microarray chip. The technique is limited to known gene sequences and is prone to background noise (Okoniewski and Miller, 2006; Royce et al., 2007), but provides a cost-effective way to measure gene transcription in high-throughput manner. It has been used to produce spatially comprehensive atlases of the human (Kang et al., 2011; Hawrylycz et al., 2012; Miller et al., 2014) and non-human primate brain [NIH Blueprint Non-Human Primate (NHP) Atlas (2009), in conjunction with ISH].

As summarized in Keil et al. (2018), there is a large number of gene expression atlases. Due to their high spatial coverage, the two most used brain-wide expression atlases are the Allen Mouse Brain Atlas (AMBA) (Lein et al., 2007) and the Allen Human Brain Atlas (AHBA) (Hawrylycz et al., 2012), both made freely available by the Allen Institute for Brain Science. The AMBA provides an extensive representation of the expression patterns of 19419 genes across the whole mouse brain, using ISH to quantify brain-wide expression patterns with the cellular resolution at each tissue slice with slices acquired every 200μm (the latter resolution depends on the section). Spatially resolved gene expression data can be further parcellated using anatomical atlases of the mouse brain (Johnson et al., 2010; Furth et al., 2018) to acquire averaged expression values through a hierarchy of brain regions defined at different resolution scales. The AHBA comprises expression measures for 21, 245 genes (depending on available annotation data) taken from 3, 702 spatially distinct post-mortem tissue samples distributed throughout the brains of six human donors (Hawrylycz et al., 2012, 2015). Both atlases have been mapped to stereotaxic space, allowing researchers to link spatial variations in gene expression to the spatial variations of a given neural phenotype (i.e., any quantifiable, spatially varying property of the brain, as measured either at the level of brain regions or pairs of regions) (Fornito et al., 2019). Other gene expression databases include both spatial (Fertuzinhos et al., 2014) and spatio-temporal (Ayoub et al., 2011; Belgard et al., 2011; Colantuoni et al., 2011; Miller et al., 2014) atlases, along with the Allen Developing Mouse Brain Atlas (2008), however most of these lack the spatial coverage of the AMBA and AHBA with only a handful regions being assessed across multiple time points. Some gene expression atlases have also been published for the macaque, using ISH and microarray (Bakken et al., 2016), and *C. elegans* (Harris et al., 2010). The latter database has been curated from published reports and contains binary entries on around 5% of the ~20, 000 genes in the full worm genome, such that the only information encoded is whether a given gene is expressed or not in a neuron.

Gene expression measures can be influenced by a number of technical and biological factors (Fraser et al., 2005; Berchtold et al., 2008; Kumar et al., 2013; Trabzuni et al., 2013). For example, the AHBA consists of data from six donor brains, each varying in characteristics, such as age at death, cause of death, sex, and ethnicity. Therefore, any analysis pooling expression measures across brains should ensure that inter-subject variability has not directly influenced the results. The analysis of gene expression measures often involves important additional processing decisions that are not applied consistently and can impact final results. For example, useful steps in processing raw AHBA data prior to analysis include (i) verifying probe-to-gene annotations; (ii) filtering genes that are not expressed above the background; (iii) selecting a representative probe when more than one probe has been used to assay a single gene; (iv) assigning tissue samples to specific brain regions in the imaging dataset; and (v) normalizing expression measures to account for inter-individual differences and outlying values. Each step requires a number of decisions, and best-practice workflows have not been established yet (Arnatkevičiūtė et al., 2019). Finally, gene expression data often shows a strong spatial autocorrelation, such that gene expression is more tightly coupled between regions that are close to each other compared to those that are spatially distant. This trend has been demonstrated in the mouse (Fulcher and Fornito, 2016), human (Richiardi et al., 2015; Krienen et al., 2016; Vértes et al., 2016; Pantazatos and Li, 2017; Arnatkevičiūtė et al., 2019) and head of *C. elegans* (Arnatkevičiūtė et al., 2018). In order to demonstrate that a putative association between regional variations in gene expression and a given neural phenotype is evident beyond this distance-dependence, potential biases introduced by the dependence can be addressed using methods ranging from simple regression (Fulcher and Fornito, 2016), partial Mantel tests (French and Pavlidis, 2011; Ji et al., 2014; Fakhry et al., 2015) or spatially constrained randomization procedures (for example, see Vértes et al., 2016; Burt et al., 2017; Seidlitz et al., 2018; Arnatkevičiūtė et al., 2019).

Brain-wide gene expression measures can be related to a brain network-level phenotype either at the level of specific brain regions (Myers et al., 2007; Rittman et al., 2016; Vértes et al., 2016; Parkes et al., 2017) or using inter-regional transcriptional coupling (Richiardi et al., 2015; Fulcher and Fornito, 2016; Arnatkevičiūtė et al., 2018; Romero-Garcia et al., 2018). Analyses of regional gene expression focus on understanding how the expression of a given gene varies across regions, and whether this variation tracks spatial variations in some other phenotype (e.g., regional gray matter volume, or number of connections). In analyses of inter-regional transcriptional coupling or correlated gene expression (CGE), each region's transcriptional profile is mapped as a vector of expression values across all genes, and these vectors are correlated between different regions, thus resulting in a region × region CGE matrix indicating the similarity between brain regions in terms of their gene expression patterns. Gene-to-gene co-expression (Eising et al., 2016; Keo et al., 2017; Negi and Guda, 2017), on the other hand, is estimated at the levels of genes (rather than regions). Each gene's expression profile across regions is summarized as a vector, and these vectors are correlated between pairs of genes, resulting in a gene × gene coexpression matrix demonstrating whether regional expression patterns for gene pairs match. Note that the term gene coexpression is sometimes used in reference to CGE. We use the current nomenclature to avoid confusion between the two.

Once a relationship between gene expression and a given neural phenotype has been established, functional groups of genes involved in driving the effect can be identified using gene set enrichment analyses (GSEA) (Subramanian et al., 2005; Irizarry et al., 2009). Since such analyses are often performed across many thousands of genes, GSEA offers a method for determining whether certain categories of genes—e.g., defined by gene ontology (GO) (Ashburner et al., 2000) or KEGG ontology (KO) (Kanehisa and Goto, 2000)—are over-represented in the set of genes showing the strongest associations. This approach allows for a functional interpretation of the results, at the expense of specificity at the level of single genes (i.e., inferences are made about functional groups of genes).

## 3. Hubs in Brain Networks

Complex behaviors require the coordination and integration of information both within and across different, functionally specialized brain regions. In primate brains, it has long been assumed that association areas, sitting atop the cortical hierarchy, and in interaction with subcortical regions, play an important role in these integrative processes (Felleman and Van Essen, 1991; Mesulam, 1998; Meyer and Damasio, 2009). Structural connectivity studies have confirmed that association areas, and regions of basal ganglia and thalamus, have high levels of connectivity, marking them as network hubs (van den Heuvel and Sporns, 2011). Artificially lesioning these nodes rapidly fragments the network, indicating that they play a vital role in network integration (Albert et al., 2000; van den Heuvel and Sporns, 2011). Moreover, both simulated node deletion and *in vivo* regional inactivation experiments demonstrate a direct relationship between a brain region's centrality and its functional impact on connected networks (Vetere et al., 2017).

Network hubs, the core elements in the network, can be defined using a range of different measures. These measures quantify distinct aspects of topological centrality, which can be defined as the capacity of a node to influence or be influenced by other nodes by virtue of its connection topology (Fornito et al., 2016). The simplest such measure is node degree, which is defined as the number of connections attached to a node. Other commonly used measures include closeness and betweenness centrality, which are both built on the premise that information in the network propagates through the most efficient route (the shortest path between regions), and thus, the centrality of any given node can be quantified by its average shortest path length (closeness), or the number of shortest paths between other nodes on which it lies (betweenness). These measures are often positively correlated across most networks, including the brain, and it is common to find a subset of nodes that score highly on most centrality measures, representing a topologically central network core (Oldham et al., 2018).

**Figure 1:**
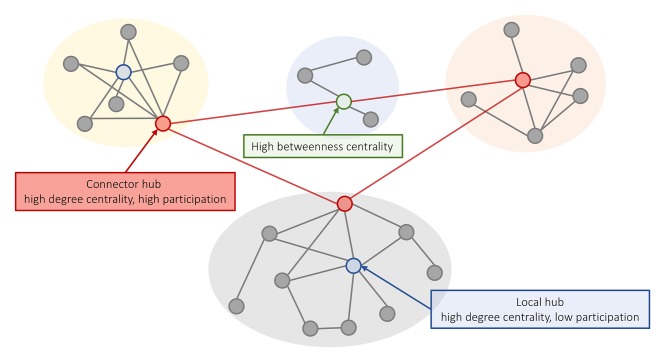
Different concepts of hubness in brain networks. A schematic representation of a modular network where nodes within a module (different background colors) show a relatively high degree of intra-modular connectivity and a low degree of inter-modular connectivity. High degree nodes can be classified into (i) local hubs (blue) that have a high degree centrality and low participation coefficient; and (ii) connector hubs (red) that have high degree and connect to nodes in other modules. Nodes with high betweenness centrality are located on shortest paths between nodes and can play an important role in linking different nodes, even if they have low degree (e.g., the green node supports communication between the yellow and orange modules).

Another way to define hubs is in relation to the modular organization of the network. Nodes within a module are densely interconnected with each other and relatively sparsely connected to nodes in other modules. Given a partition of a network into modules (e.g., Blondel et al., 2008), the integrative role of a node in the network can be characterized using the participation coefficient: a measure of connection diversity that assigns a high score to nodes with connections distributed evenly across modules. Thus, hubs defined based on the degree centrality can be further classified into “local hubs,” which connect primarily to nodes in the same module (high degree and low participation), and “connector hubs,” which connect to nodes from other modules (Figure 1) (Guimerá et al., 2012).

The interpretation of different measures of network centrality must be moderated by an appreciation of how the network has been constructed. If one investigates structural connectivity (e.g., through electron microscopy, tract tracing, or diffusion MRI) then network edges represent physical connections between network elements, and interpretation is straightforward. If one investigates functional connectivity (e.g., through electrophysiology, calcium imaging, or functional MRI), which captures statistical dependencies between physiological signals recorded at each node (Friston, 1994), the interpretation is less clear and some measures of dependence, such as the correlation coefficient, can bias the topology of the network (Power et al., 2011; Zalesky et al., 2012). Furthermore, different centrality measures make assumptions about how dynamics unfold on the network structure. For example, closeness and betweenness assume information is routed along shortest paths, which may not be a realistic model of communication in nervous systems (Goñi et al., 2014; Mišić et al., 2015; Seguin et al., 2018).

**Figure 2:**
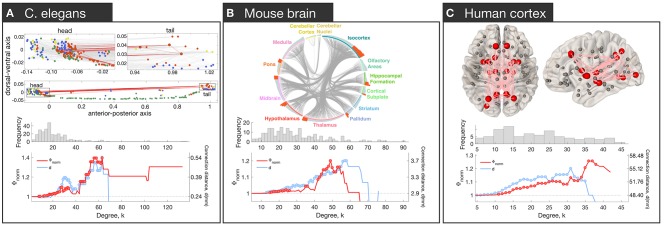
Rich club connectivity in different species. Top row: The spatial location of hubs in *C. elegans* **(A)**, mouse **(B)**, and human **(C)**. **(A)** Neurons are represented as nodes with colors corresponding to neuron type: interneurons (red), motor neurons (green), sensory neurons (blue), multimodal neurons (yellow). Hub neurons (neurons with node degree, denoted *k*, > 44) are shown as circles outlined in black. Connections between hubs are shown in red; other connections shown in gray in the upper plots. The upper part represents zoomed-in plots of the head and tail that are shown as dotted rectangles in the lower plot [adapted and reproduced from (Arnatkevičiūtė et al., 2018)]. **(B)** Meso-scale connectome of the mouse. Hub regions (regions with *k* > 44) are distributed across the whole brain and contain areas in isocortex, striatum, hippocampal formation, pallidum, thalamus, hypothalamus, midbrain, pons, and cortical subplate [adapted and reproduced from (Fulcher and Fornito, 2016)]. **(C)** Macro-scale connectome of the human brain. Hub regions (regions with *k* > 30) are shown as big red spheres while other regions as smaller gray spheres. Connections between hubs are shown in pink. Hubs are bilateral: lingual gyrus, precuneus, superior frontal gyrus, superior parietal gyrus, insula, thalamus, putamen and hippocampus; right pallidum; left caudate and lateral occipital gyrus. Middle row: Distribution of degree values across nodes. In each network, the distribution is heavy-tailed, consistent with the presence of highly connected hub nodes. Bottom row: Normalized rich-club coefficient Φ_norm_ (red) and average connection distance of hub-hub links, *d* (blue), as a function of degree (*k*) at which hubs are defined. The coefficient Φ_norm_ is defined by thresholding the network at a given level of *k*, calculating the density of connections between hub nodes (all nodes with degree > *k*), and normalizing this value by the corresponding value obtained in an ensemble of appropriately matched surrogate graphs. The normalized coefficient therefore quantifies the degree to which the density of connections between hubs exceeds chance expectations. Since the threshold to define hubs is arbitrary, the coefficient is evaluated across all possible values of *k*. A rise in Φ_norm_ at high levels of *k* is consistent with rich-club organization. Red circles indicate Φ_norm_ values that are significantly higher than an ensemble of 10,000 null networks (permutation test *p* < 0.05). Blue circles indicate where the mean connection distance between hubs is significantly greater relative to other links in the network (one-sided Welch's *t*-test; *p* < 0.05).

Brain network hubs are densely interconnected, forming a rich-club (Colizza et al., 2006). This property has been observed in the macroscale human connectome (van den Heuvel and Sporns, 2011), the mesoscale connectomes of the mouse (Fulcher and Fornito, 2016), rat (van den Heuvel et al., 2016b), cat (de Reus and van den Heuvel, 2013) and macaque (Harriger et al., 2012), and the micro-scale neuronal connectome of the *C. elegans* (Towlson et al., 2013) (Figure 2).

Given that hubs are distributed throughout the brain and involved in diverse functional systems (de Reus and van den Heuvel, 2013; van den Heuvel and Sporns, 2013; Fulcher and Fornito, 2016), dense inter-connectivity of hub nodes is thought to support efficient integration of different functionally specialized systems (van den Heuvel et al., 2012), and to increase the diversity of the brain's functional repertoire (Senden et al., 2014). This integrative capacity comes at cost, with connections between hubs extending over longer anatomical distances than other types of connections (van den Heuvel and Sporns, 2011; Harriger et al., 2012; Fulcher and Fornito, 2016; Arnatkevičiūtė et al., 2018). Hub regions also have the highest levels of resting metabolism (Vaishnavi et al., 2010; Tomasi et al., 2013) and blood flow (Liang et al., 2013). This high metabolic cost is thought to partly explain why pathology preferentially accumulates in brain network hubs across a wide range of diverse neurological diseases (Bullmore and Sporns, 2012; Crossley et al., 2014; Fornito et al., 2015).

The mechanisms resulting in the emergence of network hubs are unknown, but geometric constraints and evolutionary pressures to maximize adaptive function may play a role (Henderson and Robinson, 2014; Roberts et al., 2016; Betzel and Bassett, 2017). Whereas, generative network models based on simple geometric rules reproduce a range of statistical properties of brain networks (Ercsey-Ravasz, 2013; Henderson and Robinson, 2014; Song et al., 2014), the spatial location of hub regions cannot be explained by geometry alone (Roberts et al., 2016), suggesting an additional role for non-geometric factors in shaping the specific topology and topography of the connectome. In this context, genes may make an important contribution to shaping complex properties, such as rich-club organization. We now turn our attention to recent studies investigating the transcriptional correlates of hub connectivity by integrating connectomic data with spatially comprehensive gene expression databases across different species and scales.

## 4. The Molecular Correlates of Hub Connectivity

The first study to link transcriptional measures to the hub connectivity (Rubinov et al., 2015) combined gene expression data from the AMBA (Lein et al., 2007) with a mouse connectome inferred statistically from 461 tract-tracing studies (Oh et al., 2014). Data from these anterograde tracer injections into the right hemisphere were aggregated into a directed and weighted connectivity matrix comprising of 112 bilaterally symmetrical cortical and subcortical nodes defining edge weights as normalized connection densities and ranging over four orders of magnitude, with 53% of all possible pairs of regions showing some level of non-zero connectivity. The authors identified a subset of nodes with high degree and a high participation coefficient, indicating that they were highly connected while also being connected to nodes in diverse functional systems. Using partial least squares (PLS) (Hervé, 2010), they were able to derive a linear combination of genes whose expression levels explained 48% of the variance in nodal participation coefficient. The analysis focused on a subset of 3,380 genes form the AMBA that passed quality control criteria and were assayed in at least one additional independent experiment allowing the authors to evaluate gene expression reproducibility. The genes weighting strongly on the participation-related component were enriched for GO categories, such as learning, cognition, and memory, suggesting a link between the expression of genes related to regional variations in network participation and those implicated in cognition.

**Figure 3:**
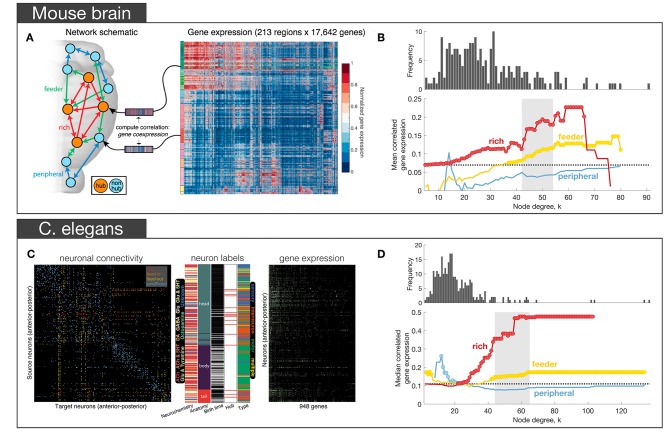
Empirical studies investigating the transcriptional properties of hub connectivity in mouse **(A,B)** and *C. elegans* **(C,D)**. **(A)** The schematic representation of different types of connections in the mouse brain: rich (connecting a hub to a hub)—red, feeder (connecting a hub to a non-hub or a non-hub to a hub)—green, peripheral (connecting a non-hub to a non-hub)—blue. Links in the connectome were categorized across this scheme. For each region, a vector of gene expression values was extracted as the corresponding row of the region in the full gene expression matrix comprising the AMBA. The matrix represents the normalized gene expression of 17,642 genes (columns) across 213 regions (rows). Gene expression profiles for each region were then used to estimate correlated gene expression (CGE) between region pairs. **(B)** Mean correlated gene expression for rich, feeder, and peripheral links as a function of node degree (*k*) where hubs are nodes with degree > *k*. The mean CGE of rich links increases at levels of *k* that coincide with a regime where evidence of topological rich-club organization is found indicating that CGE is highest for connected pairs of network hubs. The topological rich-club regime (determined from the network topology, see Figure 2A) shaded gray. Circles indicate a statistically significant increase in correlated gene expression for a given link type relative to the rest of the network (one-sided Welch-s *t*-test; *p* < 0.05) [adapted and reproduced from (Fulcher and Fornito, 2016)]; **(C)** Neuron-and-synapse connectome of *C. elegans*, reconstructed for 279 neurons using electron microscopy. Connections colored according to how they connect hubs (neurons with degree > 44) and non-hubs (neurons with degree ≤ 44): red (rich links connecting hubs), orange (feed-in links connecting a non-hub to a hub), yellow (feed-out links connecting a hub to a non-hub), blue (peripheral links connecting non-hubs). Middle: additional data acquired for each neuron, such as its: chemically secreted transmitter, anatomical location, birth time, hub status and neuronal type. Right binary gene expression profile for each of the 279 neurons (rows) across 948 genes (columns). **(D)** Median CGE for each connection type (feed-in and feed-out connections are combined and represented as feeder) as a function of node degree *k*. The topological rich-club regime (determined from the network topology, see Figure 2A) shaded gray. Circles indicate a statistically significant increase in CGE in a given link type relative to the rest of the network (one-sided Wilcoxon rank sum test, *p* < 0.05) [adapted and reproduced from (Arnatkevičiūtė et al., 2018)].

In a subsequent analysis of the Allen Institute mouse connectome, Fulcher and Fornito (2016) used a parcellation comprising 213 regions linked by 3, 063 connections (6.9% of all possible links), focusing on the right hemisphere only (where complete information on afferent and efferent connectivity was available), in combination with ISH measures of expression across 17, 642 genes in the AMBA (Lein et al., 2007). Their primary aim was to characterize how coupled patterns of gene expression between regions (i.e., correlated gene expression or CGE) relate to network topology. After confirming that the right hemisphere of the mouse connectome did indeed show evidence of rich-club organization, and that connections between hubs were both the most costly (measured by connection distance, reciprocity and weight) and central (measured using edge betweenness centrality and an alternative measure called communicability, that does rely on shortest path communication) connections of the network, they distinguished between three topological classes of connections following the work of van den Heuvel et al. (2012): (i) rich links, which connect two hubs (where hub is defined based on degree); (ii) feeder links, which connect a hub to non-hub (feed-out) or a non-hub to a hub (feed-in); and (iii) peripheral links, which connect two non-hubs (Figure 3A). Across a wide range of thresholds for defining a hub, CGE was highest for rich links, followed by feeder, and lowest for peripheral edges, with CGE showing a sharp rise at a hub threshold range that coincided with a regime in which a significant topological rich-club was observed (Figure 3B). This tightly coupled transcriptional activity between hub nodes defied a general trend in the brain where CGE between two areas decayed sharply (exponentially) as a function of their distance. That is, despite connected hubs being separated by longer anatomical distances than other pairs of regions, they showed the highest levels of transcriptional coupling (note that CGE measures were corrected for this dependence). Enrichment analysis showed that this effect was driven by genes regulating the oxidative synthesis and metabolism of ATP—the primary energetic source of neuronal communication. By comparison, an enrichment analysis comparing connected to unconnected regions (regardless of whether those connections involved hubs) found significant involvement of a large number of GO categories related to synaptic plasticity and communication, axon structure, and metabolism. These findings suggest that while genes involved in forming and maintaining synapses and axons are important for establishing a connection between two regions, the primary genomic distinction between different topological classes of connections (as defined in relation to hubs) is related to the metabolic requirements of those connections.

More recently, we found a qualitatively similar pattern of elevated CGE in rich links in the nematode *C. elegans* connectome (Arnatkevičiūtė et al., 2018). Combining electron micrograph data defining the electrochemical connectome of 279 neurons (Varshney et al., 2011) with binary gene expression profiles across 948 genes (Figure 3C) acquired from WormBase (Harris et al., 2010), we identified the same trend for CGE to be highest for rich links, followed by feeder, and then peripheral edges (Figure 3D). The involvement of metabolic genes in rich-club connectivity—as in the mesoscopic mouse connectome (Fulcher and Fornito, 2016)—could not be confirmed due to limited gene expression data in the worm, but analysis of the available data indicated that glutamate signaling and neuronal communication genes made the strongest contribution to elevated CGE for hub-hub connections (Arnatkevičiūtė et al., 2018). Leveraging the extensive additional data on neuronal phenotypes available for the worm, we found that elevated CGE for connected hubs could not be explained by a range of other properties, such as neuronal lineage distance (number of cell divisions separating pairs of neurons from a common ancestor), differences in birth time, neuronal subtype (sensory, motor, or interneuron), chemically secreted neurotransmitter, anatomical separation distance or topological module affiliation. However, the effect did seem to be driven by the fact that most hubs in the worm connectome are command interneurons, a specialized class of neurons that regulates motion. Motion is one of the more complex behaviors in the worm's repertoire, and these findings parallel evidence in primates that network hubs are primarily located in association cortices, which are thought to mediate higher-order cognition (Achard et al., 2006; Sporns et al., 2007). Thus, despite numerous differences in the data, including different gene annotation methods (~20000 ISH genes in mouse vs. ~1, 000 binary literature-curated annotations in worm), the type of the neural system (spatially continuous macroscopic mouse brain *vs* spatially separated *C. elegans* nervous system), and the orders of magnitude differences in scale, both studies demonstrated the same general pattern of increased transcriptional similarity across topologically central hub nodes.

**Figure 4:**
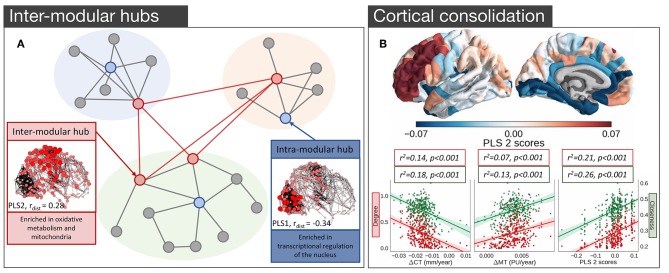
Empirical studies investigating the transcriptional properties of hub connectivity in human. **(A)** A schematic representation of the modular organization of the connectome demonstrating the key properties of inter- and intra- modular hubs based on Vértes et al. (2016). Intra-modular hubs (blue nodes) mostly connect nodes within the same module and have relatively short connection distances; characterized by the PLS1. Intra-modular hubs (red nodes) have a more diverse connectivity profile with connections extending long distances and connecting nodes from different modules; characterized by the PLS2. Size and color saturation of the nodes in the connectome corresponds to the regional scores on PLS1 (Intra-modular hub) and PLS2 (Inter-modular hub) to represent the spatial pattern of transcriptional profiles [adapted and modified from (Vértes et al., 2016)]. **(B)** Gene expression and cortical consolidation in adolescence based on Whitaker et al. (2016), (top) spatial topography of the second component from a PLS analysis corresponding to cortical consolidation during adolescence, defined as cortical shrinkage/myelination. Genes identified in this profile are related to synaptic transmission and risk to schizophrenia, among others, and are overexpressed in prefrontal areas of the cortex; (bottom) hubs in the structural covariance network experience faster rates of cortical thinning (CT) and myelination. The PLS2 gene expression profile is also significantly associated with degree, meaning that hubs are likely to over-express those genes [adapted and modified from (Whitaker et al., 2016)].

In light of the findings in both mouse and *C. elegans*, where several groups of genes implicated in cognition (Rubinov et al., 2015), oxidative metabolism (Fulcher and Fornito, 2016), and neuronal communication (Arnatkevičiūtė et al., 2018) have been identified as being related to hub connectivity, one could wonder whether the same genes are involved in the hub connectivity of the human brain. The first analysis to link gene expression and hub connectivity in humans was performed by Vértes et al. (2016), who combined resting-state fMRI (rs-fMRI) data with the high coverage genome-wide gene expression from AHBA (Hawrylycz et al., 2012). Rendering rs-fMRI data for 285 cortical regions as a binary undirected network, thresholded to retain 10% of all possible connections, they measured three different properties of each node: its within-module connectivity, its participation coefficient (between-module connectivity), and its average Euclidean distance from other nodes. PLS identified three components that collectively accounted for 37% of the total variance in nodal metrics with the first component exhibiting a positive correlation with intra-modular degree and a negative correlation with average nodal distance, corresponding to high degree nodes that mostly form short-range within-module connections. Genes positively loading on this component were enriched for GO categories related to transcriptional regulation. The second component was positively related to both the participation coefficient and average nodal distance, thus representing nodes with long connections that extend between modules, consistent with the integrative hubs of the network (Figure 4A). As seen in the analysis of the structural connectivity analysis of the mouse (Fulcher and Fornito, 2016), genes loading positively on this component were enriched in GO categories related to oxidative metabolism and mitochondrial function. These genes also showed significant over-representation for a set of 19 genes (Krienen et al., 2016) selectively enriched in the supragranular layers of the human cortex (HSE-human supragranular enriched genes) with some of those genes being implicated in the formation of corticocortical projections emanating from the higher layers of the cortex (Krienen et al., 2016). Together these findings suggest that hubs across species demonstrate conserved transcriptional properties related to their high metabolic demands.

It is well-known that the human brain undergoes an extended period of development during adolescence that is critical for brain maturation and coincides with the period of peak risk for many mental disorders (Paus et al., 2008). Some of those developmental changes particularly target hub regions (Dennis et al., 2013; Hwang et al., 2013; Baker et al., 2015; for a review see Cao et al., 2016). Whitaker et al. (2016) examined a large sample of adolescents (279, aged 14–24 years old) and found that topologically central hubs of the cortical structural covariance networks undergo an increased rate of consolidation, defined by increased cortical thinning and enhanced myelination (Figure 4B). Components of transcriptional variance that correlated with this consolidation were extracted using PLS, employing the full set of 20 737 genes from the AHBA. The first two components explaining 28% of the variance in MRI measures were related to the baseline measures of cortical thickness and myelination (PLS1), and cortical shrinkage and myelination—consolidation over time (PLS2) (Figure 4B), respectively. The PLS2 component involved contributions from genes regulating synaptic transmission and a set of genes linked to risk for schizophrenia, suggesting that deviation from the normal developmental consolidation of hub regions might manifest as an intermediate phenotype for schizophrenia (Whitaker et al., 2016), consistent with evidence that hubs are disproportionately impacted by the disease (van den Heuvel et al., 2013b; Crossley et al., 2014; Klauser et al., 2016) and that regional variations in the expression of schizophrenia risk genes track the regional variations in the magnitude of group differences in connectivity between controls and patients (Romme et al., 2017).

Importantly, this work implies that genes involved in the development of hubs, which relate to myelination and synaptic transmission, are distinct from those implicated in cross-sectional studies of adult hub connectivity, which implicate metabolic genes. In other words, the genetic mechanisms underlying the development of hub connectivity may differ from those involved in sustaining the functional role that hubs play in a mature neuronal system. The further development of brain-wide atlases of developmental changes in gene expression will help shed light on how such differences can be leveraged to gain insight into the development of different brain disorders.

## 5. Conclusions and Further Directions

Brain-wide gene expression atlases provide exciting opportunities to link different scales of brain organization. At the same time, integrating such data with connectomic measures poses challenges. Given the nascence of this field, no standardized data processing pipelines have been developed, with widespread inconsistencies in processing of the same transcriptional data across studies (Arnatkevičiūtė et al., 2019) complicating direct comparison between findings, even within the same species. Nonetheless, the available studies—conducted in diverse species and using different measures of brain connectivity and gene expression acquired at different resolution scales—point to a conserved transcriptional signature of hub connectivity related to genes regulating neuronal communication and metabolism, consistent with the high centrality and metabolic cost of hub regions (Bullmore and Sporns, 2009).

One limitation affecting the human data is that the gene expression measures are derived from bulk tissue samples. The cellular composition of these samples can influence measured gene expression patterns, such that two samples can differ in their transcriptional properties simply due to the differences in the density of distinct cell types. Single-cell transcriptomics is able to provide precise gene expression measurements in individual cells, thus resolving cell-specific transcriptional profiles. While scRNA-seq is not currently feasible for the whole human brain, the expression profiles of specific cell groups in the adult (Johnson et al., 2015; Hu and Wang, 2017; Picardi et al., 2017) and developing brain (Zhong et al., 2018) are being characterized.

These limitations notwithstanding, the consistency of results considered here—often identified through unbiased, data-driven techniques—demonstrate the potential utility of brain-wide transcriptomic measures in yielding biologically meaningful insights to otherwise abstract graph-theoretical structures, such as hubs and other neural phenotypes. With the availability of new resources and developments in neuroimaging, the combination of such data across resolution scales offers a promising way forward for uncovering the molecular mechanisms that drive the large-scale organization of the connectome.

## Author Contributions

AA wrote and edited the manuscript. BF and AF provided feedback, structured, and edited the manuscript. All the authors planned the structure of the manuscript.

### Conflict of Interest Statement

The authors declare that the research was conducted in the absence of any commercial or financial relationships that could be construed as a potential conflict of interest.
